# Human papillomavirus genotypes in cervical cancer and vaccination challenges in Zimbabwe

**DOI:** 10.1186/1750-9378-9-16

**Published:** 2014-05-13

**Authors:** Nyasha Chin’ombe, Natasha L Sebata, Vurayai Ruhanya, Hilda T Matarira

**Affiliations:** 1 Department of Medical Microbiology, University of Zimbabwe, A178, Avondale, Harare, Zimbabwe; 2 Department of Chemical Pathology, University of Zimbabwe, A178, Avondale, Harare, Zimbabwe

## Abstract

Cervical cancer is one of the major causes of morbidity and mortality in women in Zimbabwe. This is mainly due to the high prevalence of high-risk human papillomavirus (HPV) genotypes in the population. So far, few studies have been done that showed the presence of high-risk genital HPV genotypes such as 16, 18, 31, 33, 52, 58 and 70 in Zimbabwean women with cervical cancer. The prevalence of HPV DNA in women with cervical cancer has been shown to range from 63% to 98%. The high-risk HPV 16, 18, 31, 33 and 58 were the most common genotypes in all the studies. The introduction of the new HPV vaccines, HPV2 and HPV4, which protect against HPV genotypes 16 and 18 into Zimbabwe is likely to go a long way in reducing deaths due to cervical cancer. However, there are few challenges to the introduction of the vaccines. The target population for HPV vaccination is at the moment not well-defined. The other challenge is that the current HPV vaccines confer only type-specific (HPV 16 and 18) immunity leaving a small proportion of Zimbabwean women unprotected against other high-risk HPV genotypes such as 31, 33 and 58. Future HPV vaccines such as the nanovalent vaccine will be more useful to Zimbabwe as they will protect women against more genotypes.

## Introduction

Human papillomaviruses (HPVs) are a group of highly ubiquitous double-stranded circular DNA viruses that infect cutaneous and mucosal surfaces [1]. They are divided into low-risk and high-risk genotypes [2]. The main low-risk genotypes are HPV genotypes 6, 11, 40, 42, 43, 44, 54, 61, 70, 72, 81 and the main high-risk groups are HPV genotypes 16, 18, 31, 33, 35, 39, 45, 51, 52, 56, 58, 59, 68, 73, 82. It is now well established that infection of the cervical tissue with the high-risk HPV genotypes is a necessary cause of cervical cancer in women [3-5]. At the moment, public knowledge of HPV and cervical cancer is still lacking in Zimbabwe. The lack of knowledge on cervical cancer and HPV was also found during our previous cervical cancer needs assessment study in 2 provinces of Zimbabwe a few years ago [6].

### The burden of cervical cancer in Zimbabwe

Zimbabwe is a low-income country in Southern Africa and is bordered by Botswana, South Africa, Zambia and Mozambique. According to the latest population census, Zimbabwe has a population of 12,5 million people of which approximately half are female. Nationally, the Ministry of Health and Child Care has the primary responsibility of providing health care services to the majority of the population, including maternal and reproductive health services such as prevention and treatment of cervical cancer. Worldwide, cervical cancer is the second most common malignancy in women [7]. Almost 80% of cervical cancer cases are found in low income level countries [8,9]. In most African countries, cervical cancer is a leading cause of death in women [10]. The age-standardised incidence rates of cervical cancer in Africa ranges from 42.7 to 12.1 per 100 000 women [11]. In Zimbabwe, cervical cancer is an important reproductive health problem and is a major cause of mortality and morbidity in women than any other cancers [12]. It accounts for 32.2% of all female cancers and is the most common [12]. The age-adjusted incidence rate of cervical cancer in Zimbabwe is estimated at about 52.1 per 100,000 women and age-adjusted mortality rate is 43.1% [13]. The exact number of women suffering and dying from cervical cancer is likely to be higher than those quoted by the Zimbabwe National Cancer Registry as there are many women dying of the disease in the rural areas and are not reported. Some of the women who die have never attended a health facility up to the time of their death.

### HPV genotypes in cervical cancer in Zimbabwe

In Zimbabwe, a number of studies have been done to evaluate high-risk HPV genotypes present in women with cervical cancer. Firstly, a cross-sectional study (n = 119) was done to investigate the prevalence of low-risk and high-risk HPV genotypes in women with cervical cancer at two referral hospitals, Harare Central and Parirenyatwa Hospitals in Harare in Zimbabwe [14]. Using the Hybrid Capture assay, HPV DNA was detected in 63% of the cervical swabs. The prevalence of any of the high-risk HPV genotypes (16, 18, 31, 33, 35, 45, 51, 52 and 56) was 51%. The low-risk HPV genotypes (6, 11, 42, 43 and 44) were detected in 26% of the samples. In this study, dual infection with both the low-risk and high-risk HPV genotypes was found in 14% of the cases. This was the first study to show an association of certain HPV genotypes with cervical cancer in Zimbabwe. In a second study in Harare, the presence of HPV genotypes in cervical swabs and urine collected from Zimbabwean women (n = 43) who had cervical cancer was conducted using restriction fragment length polymorphism-polymerase chain reaction [RFLP-PCR] [15]. Of all the women studied, 98% and 72% had HPV DNA in cervical swabs and urine samples respectively. In this study, the high-risk HPV 16 was the most prevalent genotype and was found in 59% of the cervical samples. HPV 33 was found in 31% of the samples followed by HPV 18 (14%), HPV 31 (2%) and HPV 58 (2%). It was also noted in this study that the HPV genotypes found in the urine samples were the same as those found in the cervical samples. Of the 28 urines samples typed, 61%, 16%, 13% and 3% had HPV 16, 33, 18 and 31 genotypes respectively. According to this study, HPV 33 was the second commonest genotypes found in Zimbabwe and is not covered by the current two HPV vaccines on the market.

In a third study with 98 Zimbabwean women with cervical cancer in Harare, HPV genotypes were also investigated [16]. The HPV DNA was detected in 97% of the samples. Of the cases, HPV 16, 33, 18 and 31 genotypes were found in 61%, 39%, 18% and 4% of the samples respectively. HPV 35 and 58 were also found in some samples. It was also noted in this study that some patients who were dually infected with both HPV 16 and HPV 33 and most of them were young (10–13 age-group).

Another study was conducted to determine cervical HPV incidence in a cohort of 2040 HIV-negative Zimbabwean women [17]. The prevalence of HPV genotypes, 58, 16, 70, 18, 33, 53, 52 and 31 was 5.0%, 4.7%, 2.4%, 2.3%, 2.0%, 1.8%, 1.3% and 1.3% respectively [17]. This study showed that the high-risk HPV 58 was more common than genotype 16 or 18 that are covered by the current HPV vaccines. Overall, these few studies mentioned above highlight the need to have HPV vaccines in Zimbabwe that will cover all most prevalent genotypes.

### Risk factors of cervical cancer in Zimbabwe

It is now generally agreed that persistent infection by high-risk HPV genotypes is the major cause of cervical cancer [18]. There are several risk factors that universally predispose women to acquiring high-risk HPVs that may cause cervical cancer [19]. The factors include multiparity, early age of first intercourse, multiple sexual partners, poor genital hygiene, use of oral steroid contraceptives, cigarette smoking, some dietary factors and infection with other sexually transmitted pathogens such as *Chlamydia trachomatis* and herpes simplex virus which cause chronic cervicovaginal inflammation [20-23]. In addition to the above factors, there are other cofactors such as host genetic factors and immunodeficiency that are considered to be also important for development and progression of cervical cancer [20,24]. Other factors such as race and ethnicity are also important risk factors of cervical cancer [25]. African women are at a higher risk of developing cervical cancer than their Caucasian counterparts [22,26]. Our research group at the University of Zimbabwe also previously carried out a national cervical cancer needs assessment study of Zimbabwean (African) women in two provinces [6]. Some of the risk factors of cervical cancer were investigated. From the study, it was noted that 76% of cervical cancer in Zimbabwe presented late at health care facilities. The high percentage of women (44%) in the study did not use contraceptives such as condoms that can protect against HPV acquisition. Only 4% of married couples used barrier/condoms. The mean parity was 5 children. Sexual activity in Zimbabwean girls was found to start at 15 and this was likely to predispose them to HPV infection at an early age. From the study, it was also noted that knowledge of cervical cancer and HPVs was very low in the population. The study highlighted some of the risk factors that predisposed women to HPV infection and the gaps in prevention of cervical cancer in Zimbabwe.

### HPV vaccination and challenges in Zimbabwe

Two HPV vaccines have recently been developed to prevent cervical cancer in women [27,28]. In Zimbabwe, considerations are now being made to vaccinate girls and women against HPV using the two recently licensed subunit vaccines, HPV2 and HPV4. The vaccines are now available in the private sector and will be introduced into the public health institutions soon. HPV2 is a bivalent vaccine that prevents mainly HPV-16 and HPV-18, while HPV4 is a quadrivalent vaccine that prevents infection by HPV-16, HPV-18, HPV 6 and HPV 11 genotypes. The two vaccines have been shown to be effective and efficacious in preventing cervical cancer caused by HPV 16 and 18 genotypes and genital warts caused by HPV 6 and 11 genotypes [29-32]. Although the two vaccines can be very helpful in reducing cervical cancer mortality in the long-term, their introduction in Zimbabwe is likely to meet several challenges. Since the two vaccines only target HPV genotypes 6, 11, 16, and 18, the vaccines may not be effective in protecting against other HPV genotypes that are common in Zimbabwe and cause cervical cancer. According to studies mentioned above, the high-risk HPV genotypes 31, 33 and 58 were also found in a substantial proportion of women with cervical cancer [14-16]. Women infected with such genotypes will not be fully protected from getting cervical cancer by the current vaccines. This challenge has also been observed in other African countries such as Zambia, Cameroun, Mozambique and Senegal where other non-16 and 18 genotypes were prevalent [33-36]. Although cross-protection against multiple genotypes by the current HPV vaccines may occur, it is not absolute [37-39]. The challenge therefore confirms the importance of developing HPV vaccines for genotypes common in African women. The new HPV nanovalent vaccine still in clinical trials [40] will be more useful in Zimbabwe and other Sub-Saharan African countries since it may protect against more HPV genotypes (6/11/16/18/31/33/45/52/58).

There are other HPV vaccination challenges in Zimbabwe. The target group for HPV vaccination has not yet been defined. Generally, girls should be targeted for HPV vaccination before the onset of sexual activities [41]. No studies on HPV infection in girls or boys of this age-group have been done in Zimbabwe. Epidemiological studies are still required to ascertain the HPV prevalence by age and sex in Zimbabwe. The other challenge to HPV vaccination in Zimbabwe is the prohibitive cost ($180 - $300 for 3 doses in the private health care sector) of the vaccines to most people. Although the cost is high, the vaccines are readily available in the private sector. In the public health care sector, government or donor funding may have to subsidize the vaccines if more girls and women are to be reached. The funding for an HPV Vaccine Demonstration Project has been sourced from donors such as Global Alliance for Vaccine and Immunization (GAVI), UNICEF, WHO and UNFPA. The HPV vaccines will be introduced to two districts of Marondera and Beitbridge and will be targeted to adolescent girls aged 10 years. The government of Zimbabwe will assist with funding for the implementation of the project. It is hoped that the demonstration project will provide data that can be used for future national HPV vaccine rollout.

In Zimbabwe, as in most developing countries, there are no national cervical cancer screening or HPV testing programmes. Although 80% of all cervical cancers occur in developing countries, only about 5% of the at-risk women are screened for cervical cancer [42]. Screening of cervical cancer using cytology, visual inspection as well as HPV testing can reduce the high mortality in women in Zimbabwe. It has been suggested that HPV testing and cytology together would increase the chance of detecting cervical cancer by about 35% [43]. No services for HPV testing in public and private sector are available. Cervical cancer prevention and control through screening and HPV vaccination should therefore be a national priority if women are to be saved from dying from cervical cancer, a preventable disease.

## Conclusion

Cervical cancer caused by high-risk HPV genotypes accounts for substantial morbidity and mortality in Zimbabwe. The government is now planning to introduce HPV vaccines in girls in 2014 and the potential usefulness of these vaccines cannot be underestimated. Although there are few challenges, the HPV vaccines are likely to go a long way in reducing the prevalence of cervical cancer in Zimbabwean women.

## Competing interests

The authors declare no competing interest.

## Authors’ contributions

All the authors contributed to the writing of the manuscript. All authors read and approved the final manuscript.
